# Diagnostic Accuracy of a Commercial ELISA for Detecting Antibodies Against *Strongyloides stercoralis* in Dried Blood Spots: A Field Study in Puerto Iguazú, Argentina

**DOI:** 10.3390/pathogens15101049

**Published:** 2026-10-02

**Authors:** Rubén O. Cimino, Ernesto Candela Senti, Tamara García, Sofía Ciotta, Elvia Nieves, Carolina Goizueta, Miguel Martínez-Lacalzada, María Victoria Periago

**Affiliations:** 1 Instituto de Investigaciones de Enfermedades Tropicales (IIET), Facultad Regional Orán, Universidad Nacional de Salta, Salta A4400, Argentina; tamygarcia39038@gmail.com (T.G.); sciotta.unsa@gmail.com (S.C.); 2 Cátedra de Química Biológica, Facultad de Ciencias Naturales, Universidad Nacional de Salta, Salta A4400, Argentina; elviaesternieves@gmail.com; 3 Consejo Nacional de Investigaciones Científicas y Técnicas (CONICET), Buenos Aires C1425FQB, Argentina; 4 Facultad de Farmacia y Tecnología Farmacéutica y Parasitología, Universidad de Valencia, 46010 Valencia, Spain; erneinstein666@hotmail.com; 5 Fundación Mundo Sano, Buenos Aires C1055ABX, Argentina; cgoizueta@mundosano.org; 6 Instituto de Salud Global, 08036 Barcelona, Spain; miguellacalzada@gmail.com

**Keywords:** *Strongyloides stercoralis*, serology, dried blood spots, diagnosis

## Abstract

Background: *Strongyloides stercoralis* is frequently underdiagnosed due to the limited sensitivity of stool-based methods. Serology improves detection, but venous blood collection and cold-chain requirements complicate field implementation. Dried blood spots (DBS) offer a practical alternative for large-scale surveys. This study aimed to evaluate the diagnostic accuracy of DBS-based serology and compare its performance with conventional ELISA. Methods: A cross-sectional study was conducted in peri-urban communities of Puerto Iguazú, Misiones, Argentina. Stool samples were examined using sedimentation–concentration and Baermann techniques. Venous blood on DBS was analyzed using a commercial ELISA (cELISA) and an in-house NIE-ELISA. Diagnostic accuracy was assessed using a primary reference standard. Agreement between serum and DBS was evaluated using kappa statistics, and ROC curves were generated for paired samples. Results: DBS cELISA showed the highest sensitivity (89.1%), followed by serum cELISA (81.1%) and in-house NIE-ELISA (71.7%). Concordance between sera vs DBS was substantial for cELISA (κ = 0.68) and fair for NIE-ELISA (κ = 0.21). ROC analysis showed comparable AUCs for cELISA using sera (0.85) and DBS (0.83), whereas in-house NIE-ELISA performance declined with DBS (AUC = 0.61). Conclusion: DBS-based cELISA demonstrated high diagnostic accuracy and strong agreement with sera, supporting its use as a field-adapted tool for strongyloidiasis surveillance. DBS serology represents a feasible and reliable alternative for epidemiological studies in resource-limited settings.

## 1. Introduction

Strongyloidiasis is caused by the soil-transmitted helminth *Strongyloides stercoralis*, a parasite whose global prevalence remains underestimated due to the limited sensitivity of conventional parasitological techniques, including the Baermann method, sedimentation–concentration procedures, and agar plate culture [1]. Despite these limitations, these methods remain reference standards in diagnostic accuracy studies. Accurate prevalence estimates are essential for guiding control strategies, particularly in endemic regions where *S. stercoralis* infection may lead to severe complications in immunosuppressed individuals [2]. Serological methods, among them using recombinant NIE antigen, have demonstrated superior sensitivity compared to stool-based diagnostics and are therefore well suited for epidemiological surveys [3,4,5,6]. However, their implementation in field studies is often hindered by challenges related to serum collection, preservation, and transport, especially in remote settings. Dried blood spots (DBS) collected on filter paper offer a practical alternative that mitigates these logistical constraints. DBS have proven effective for the serodiagnosis of various infectious diseases and facilitate large-scale sero-epidemiological investigations [7].

Although DBS have been used in only a limited number of studies on strongyloidiasis, previous evidence supports their feasibility for seroepidemiological screening. Early work in Brazil demonstrated the use of DBS with immunofluorescence assays [8]. Subsequently, DBS were applied to measure antibody responses using NIE-ELISA in an Australian population; however, that study did not compare DBS results with conventional serum-based serology [9]. A key advancement was provided by [10] who directly compared DBS-based ELISA with conventional serum ELISA in school-aged children in Ecuador and reported excellent agreement between the two methods (κ = 0.92). Their findings support the use of DBS as a reliable and field-adapted tool for *S. stercoralis* serosurveillance. Given the need for accessible diagnostic strategies in resource-limited settings, evaluating the performance of DBS-based serology relative to standard parasitological and serological methods is essential. The objective of this study was to assess the diagnostic performance of DBS serology and compare it with conventional serum ELISA and parasitological testing for the screening of *S. stercoralis* infection from an endemic area in Argentina.

## 2. Materials and Methods

### 2.1. Study Design and Setting

We conducted a cross-sectional study in Puerto Iguazú, Misiones, Argentina, an area that includes peri-urban and rural communities with a documented high prevalence of intestinal parasites, including *S. stercoralis*. The serum sample panel used in this study has been previously described and analyzed using parasitological methods such as reference tests (Baermann and/or sedimentation) [11,12].

**Figure 1:**
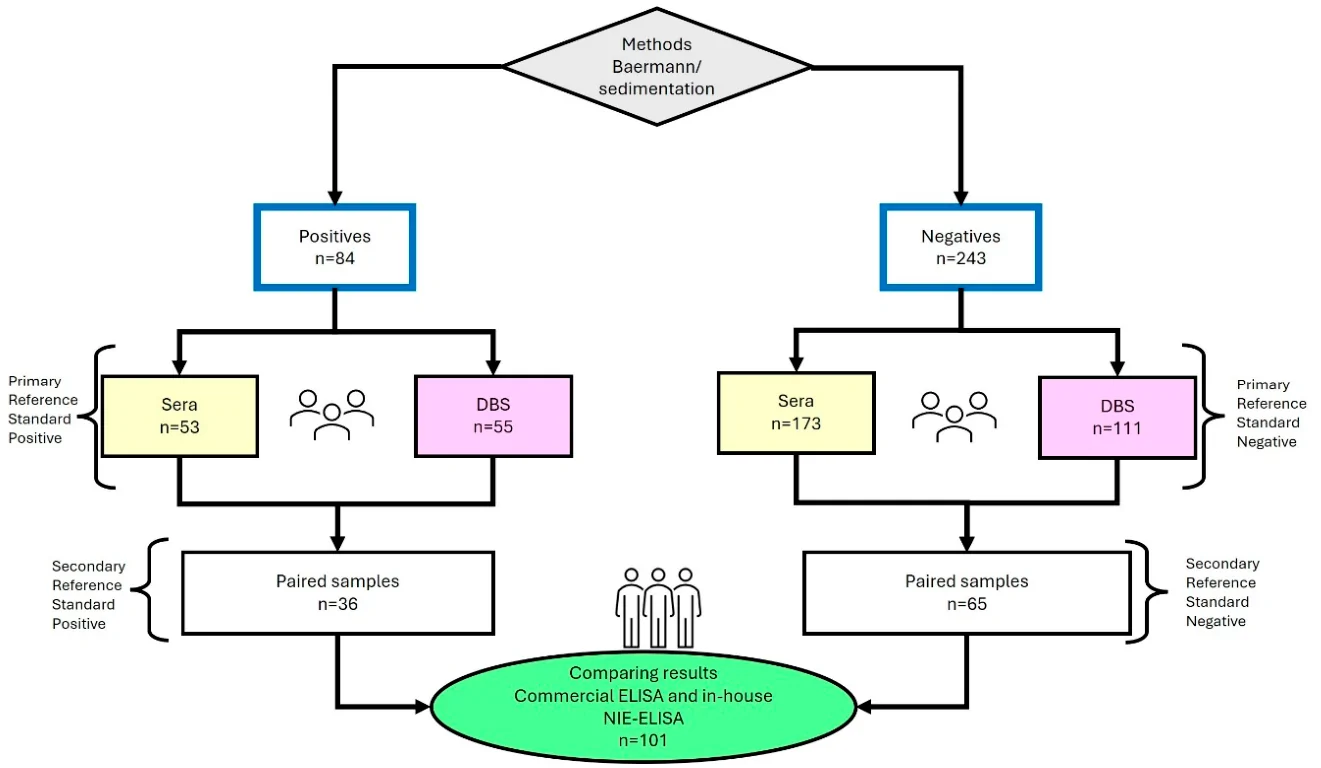
Flow chart of samples included in the study.

Following this initial classification, serum and dried blood spot (DBS) samples from the same individuals were analyzed to assess matrix compatibility (serum vs. DBS) and to compare the performance of two serological assays: a commercial ELISA and an in-house NIE-ELISA. Thus, while stool examination defined the study’s initial framework, the main analytical focus of this manuscript lies in evaluating serological performance and matrix behavior, rather than in parasitological diagnosis. Results are reported following the STARD (Standards for Reporting Diagnostic Accuracy) guidelines [13]. We used two reference standards: the PRS included all serum and DBS samples classified as positive or negative by Baermann/sedimentation, while the SRS included only paired serum–DBS samples, both positive and negative, to allow direct matrix comparison (Figure 1).

#### Size and Sample Collection

A total of 327 stool samples were collected between April and July 2023 for parasitological analysis. We obtained approximately 5 mL of venous blood from each participant in serum tubes via venipuncture (*n* = 226), and DBS samples (TropBio Filter Paper Disk for Blood Collection, manufactured by Cellabs Pty Ltd. for TropBio Pty Ltd, (Sydney, Australia.) via finger-pricking (*n* = 166), for serological antibody detection of *S. stercoralis*.

### 2.2. Study Laboratory Procedures

DBS were collected onto filter paper cards, stored in Ziploc bags containing silica beads as a desiccant, and maintained at −20 °C in the field laboratory. They were then transported to the laboratory at the National University of Salta, Salta province. The DBS were assayed between 5 and 11 months after collection, with a total storage time of up to 11 months from collection to assay completion.

**Figure 2:**
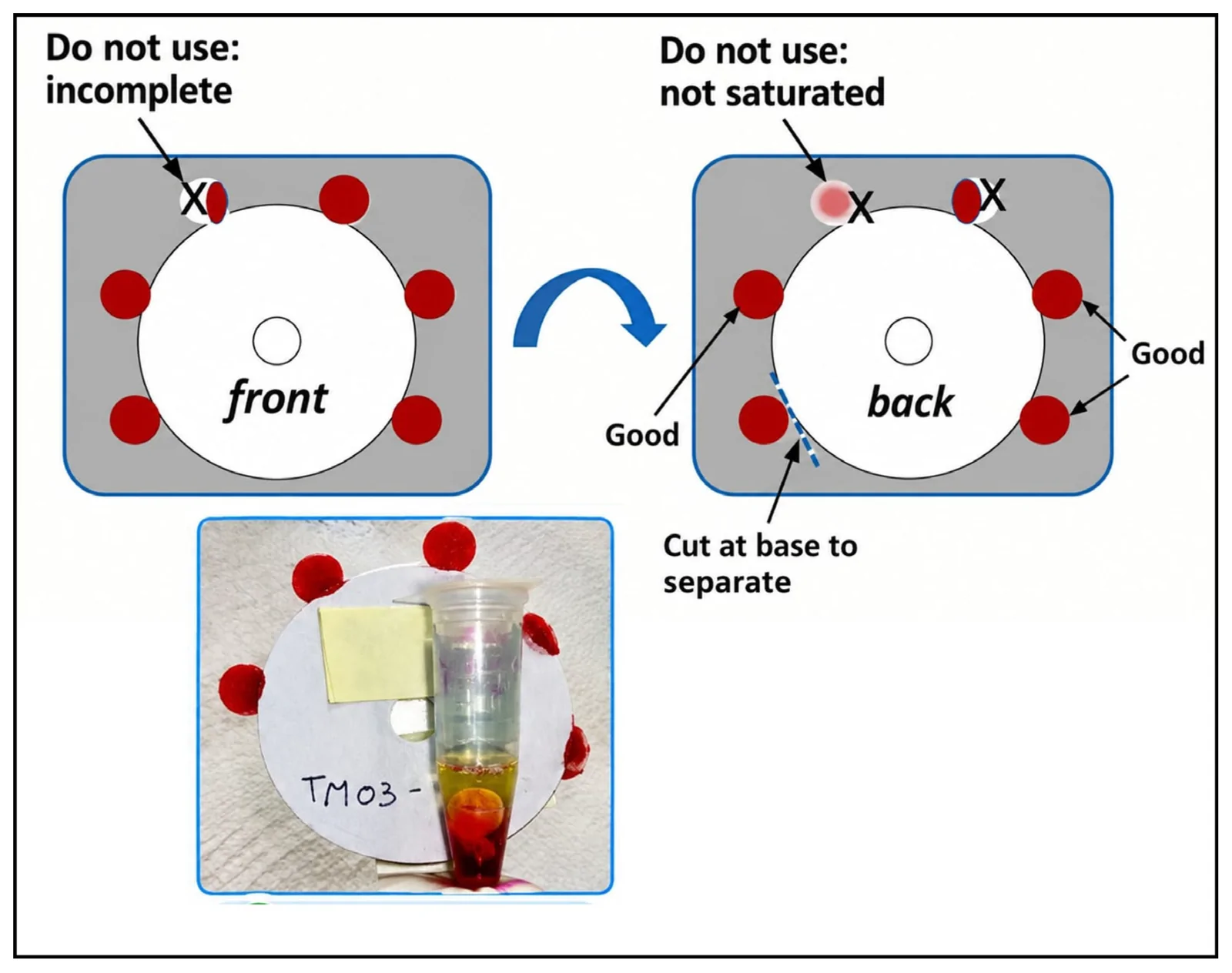
Visual guide for DBS quality and processing. The upper panels show criteria for DBS acceptance, distinguishing fully saturated spots from incomplete or non-saturated spots that must be discarded. The lower panel displays a collected DBS card ready for laboratory processing.

The presence of IgG antibodies against *S. stercoralis* (anti-NIE antibodies) was determined in the eluted DBS samples using three alternatives:

(1) Sample dilution in distilled water (TropBio protocol): We followed the manufacturer’s protocol. Each filter paper “ear” (i.e., one fully saturated circular spot) contained 10 µL of blood. For DBS elution, distilled water was added to a 2.0 mL microfuge tube to prepare 1:100 and 1:200 dilutions. The filter paper was left immersed overnight at 4 °C and then centrifuged for 5 min at 10,000× *g*; the supernatant was then collected with a pipette for testing (Figure 2).
(2) Sample dilution in commercial ELISA buffer (cELISA): We used the sample dilution buffer from the commercial test kit, following the manufacturer’s instructions and the laboratory’s TropBio protocol. We prepared and evaluated samples at 1:100 and 1:200 dilutions.
(3) Sample dilution in buffer (in-house NIE-ELISA): We used a sample dilution buffer of PBS-Tween 20 and skimmed milk. We prepared and evaluated samples at 1:100 and 1:200 dilutions.

#### 2.2.1. Standardization of DBS Processing Methods

Our working group conducted several experiments to evaluate the reproducibility of eluted DBS samples. Based on the existing literature, different eluents, including distilled water, PBS-milk, and the diluent of the cELISA, were assessed. Distilled water, recommended by the TropBio protocol, showed 100% nonspecificity when analyzed with cELISA (NovaLisa, NovaTec Immunodiagnostica, Dietzenbach, Germany) and in-house NIE-ELISA. The most effective options were the cELISA diluent for DBS samples analyzed by cELISA and PBS-milk (1%) for those analyzed by in-house NIE-ELISA (Figure S1).

The eluted samples were tested at dilutions of 1:100 (recommended by the manufacturer) and 1:200, with the aim of defining the optimal dilution that would allow distinguishing negative from positive samples. For both ELISA tests, the 1:200 dilution was selected because it exhibited lower background noise.

The DBS (dilution 1:200) and serum (dilution 1:100) samples were analyzed using the cELISA and the in-house NIE-ELISA assay. The cELISA is an indirect ELISA coated with NIE recombinant antigen. We followed the manufacturer’s instructions and interpretation of the results (Units %): positive (absorbance/control cut-off ≥11), equivocal (absorbance reading/control cut-off 9 to 11), and negative (absorbance reading/control cut-off <9). The test and interpretation of the results were performed using the Biotek instrumental ELx800 (Winooski, VT, USA). All samples were analyzed in duplicate. The in-house NIE-ELISA also uses NIE recombinant antigen to detect *S. stercoralis* infection. The enzyme-linked immunosorbent assay with rNIE (NIE-ELISA) was performed as previously described, with minor modifications [14].

#### 2.2.2. Statistical Analysis

Diagnostic accuracy (sensitivity, Se; and specificity, Sp.) was estimated using a primary reference standard based on sedimentation and Baermann methods, and all parameters were reported with their corresponding 95% confidence intervals (95% CI). Agreement between diagnostic methods was assessed using the Kappa index (κ), interpreted according to standard criteria: <0.20 (poor), 0.21–0.40 (fair), 0.41–0.60 (moderate), 0.61–0.80 (good), and 0.81–1.00 (very good). Area under the curve (AUC) ROC analyses for paired serum and DBS samples were performed in RStudio v.2026.04. Build 526 (pROC package), testing the null hypothesis AUC = 0.5 to evaluate discrimination capacity across matrices. The analysis was performed using Jamovi software version 2.6.44.0 [15].

Paired quantitative comparisons between sera and DBS were evaluated using the Wilcoxon matched-pairs signed-rank test. Median paired differences and their 95.4% confidence intervals were analyzed. Agreement between matrices was further assessed using Bland–Altman analysis, reporting the bias (mean difference) and the 95% limits of agreement (LoA), calculated as bias ± 1.96 × SD of the paired differences. The analysis was performed using GraphPad Prism version 8.0.1 for Windows (GraphPad Software, San Diego, CA, USA, www.graphpad.com, 25 May 2020).

### 2.3. Ethical Considerations

This study was approved by the Bioethics Committee of the Ministry of Public Health of the Government of the Province of Misiones, Argentina (N°28-06-2018, approval date: 28 June 2018), with its appropriate written assent and consent forms [12]. The same committee approved an addendum including the current serological techniques (Date 25 January 2023). Each participant signed an informed consent form after being informed about the goal of the study and the sampling procedures.

## 3. Results

### 3.1. Accuracy Using Primary Reference Standard (PRS)

The overall accuracy of the index test using the PRS for sensitivity (subjects with positive fecal results) and specificity (subjects with negative fecal results) is summarized in Table 1. The most sensitive test was the cELISA using DBS, with a sensitivity of 89.1%. In-house NIE-ELISA sensitivity reached 71.7%, and the lowest sensitivity was for DBS NIE-ELISA at 50.9%.

### 3.2. Accuracy Concordance Using Secondary Reference Standard (SRS)

Agreement between sera and DBS was assessed in the subset of paired samples (*n* = 101) that corresponded to the SRS (Figure 1). A total of 68.2% (30/44) of the sera positive by cELISA were also positive by in-house NIE-ELISA.

The commercial ELISA showed good concordance between matrices (κ = 0.68; 95% CI: 0.54–0.82), whereas the in-house NIE-ELISA demonstrated only fair agreement (κ = 0.21; 95% CI: 0.02–0.41).

**Figure 3:**
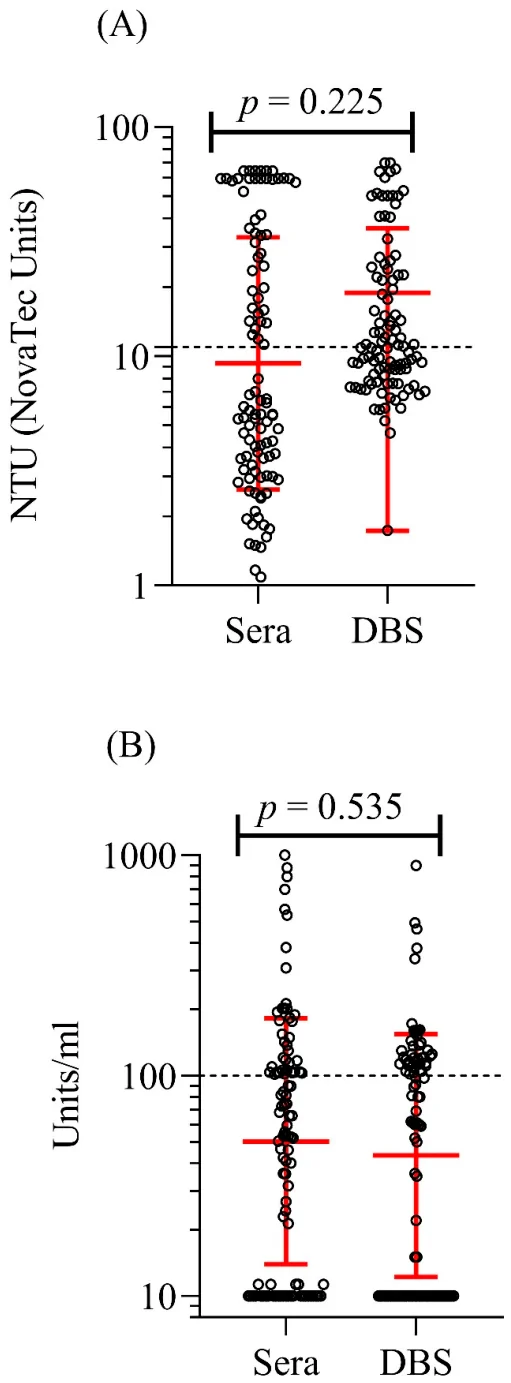
Distribution and median optical density (OD) values measured by cELISA (**A**) and in-house NIE-ELISA (**B**) across 101 paired samples. Horizontal dashed lines for each technique indicate cut-offs. Error bars (red line) represent the interquartile range (IQR), defined as the 25th–75th percentiles.

Paired quantitative comparisons were performed using the Wilcoxon matched-pairs signed-rank test. For the cELISA, the test indicated no significant difference between sera and DBS (*p* = 0.225), with a median difference of −3.33 units [95.4% CI: −4.35 to −1.93]. Bland–Altman analysis showed a bias of 0.62 units and limits of agreement ranging from −33.0 to 34.3 (Figure 3A).

For the in-house NIE-ELISA, the Wilcoxon test also indicated no significant difference between matrices (*p* = 0.535), with a median difference of 0.00 units [95.4% CI: −10.85 to 31.08]. Bland–Altman analysis showed a bias of 24.6 units and limits of agreement ranging from −270.3 to 319.4 (Figure 3B).

### 3.3. Estimation of AUC ROC Curves

**Figure 4:**
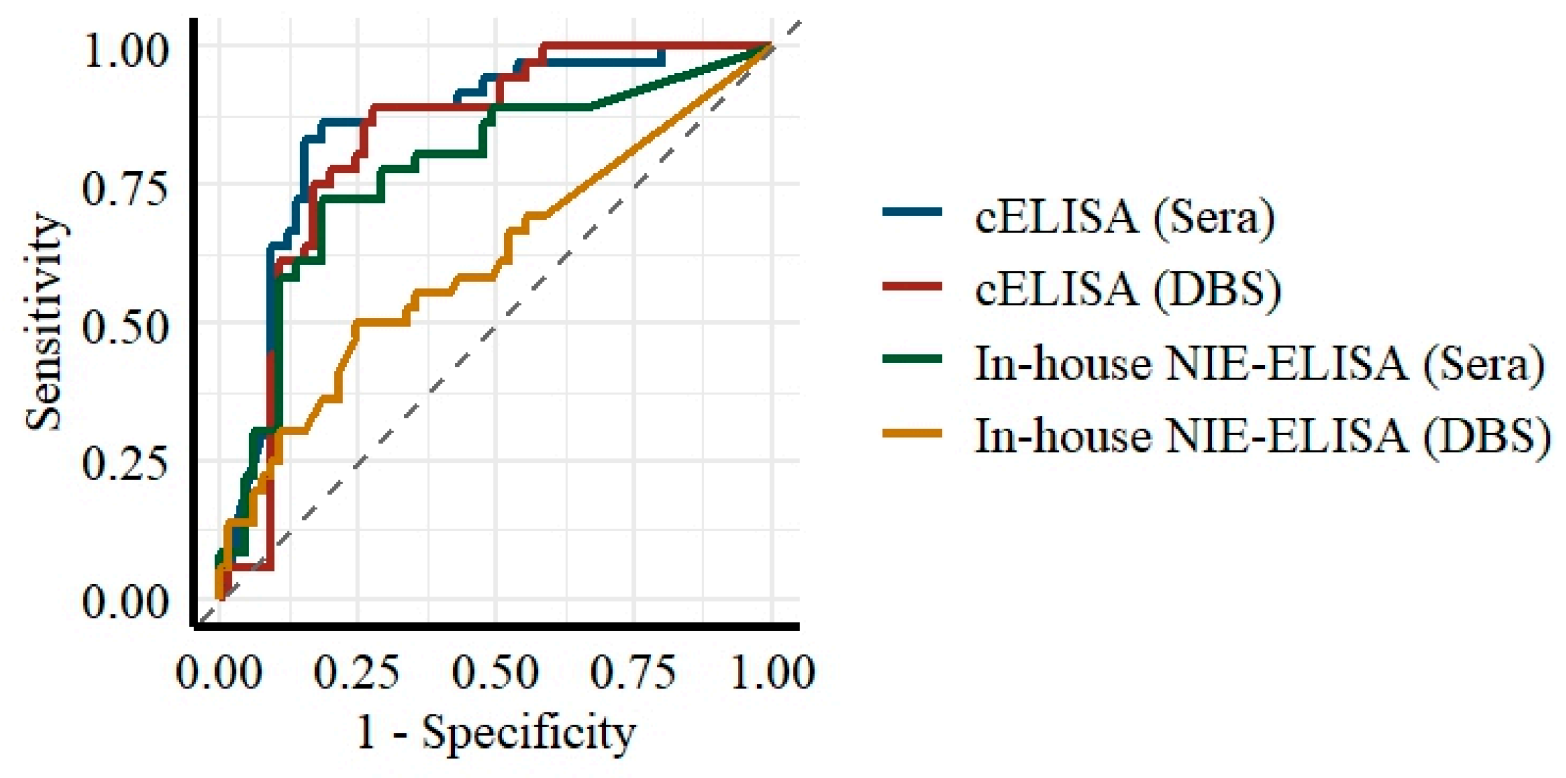
Comparison of area under the ROC curve using paired samples, secondary reference standard (n = 101).

The cELISA demonstrated the highest diagnostic performance, with an AUC of 0.85 [95% CI 0.78–0.93] when using sera, and a comparable AUC of 0.83 [95% CI 0.75–0.91] when using DBS. For the in-house NIE-ELISA, sera also outperformed DBS, yielding an AUC of 0.78 [95% CI 0.68–0.88] compared to 0.61 [95% CI 0.50–0.73]. Pairwise comparisons showed no significant difference between serum and DBS for the cELISA (*p* = 0.4325), whereas the performance gap for NIE-ELISA was statistically significant (*p* = 0.019), indicating a marked reduction in diagnostic accuracy when DBS was used for this assay (Figure 4 and Table 2).

## 4. Discussion

This study provides evidence supporting the use of DBS as a practical and reliable matrix for serological screening of *S. stercoralis* in field conditions. Working in peri-urban communities of Puerto Iguazú—where logistical constraints, environmental humidity, and limited laboratory infrastructure challenge conventional diagnostics—we demonstrated that DBS-based serology can achieve diagnostic performance comparable to serum-based assays when paired with an optimized commercial ELISA.

The cELISA showed consistently high sensitivity, with DBS even outperforming sera under the PRS. Although the DBS cELISA showed higher sensitivity than the sera cELISA, this difference should be interpreted cautiously. First, the number of true positives identified by coproparasitology was relatively small (*n* = 53), and the absolute difference between matrices was limited to only six samples (Table 1). Second, DBS frequently exhibited higher background optical density values, which may artificially elevate readings and increase the likelihood of false positives (See Figure S1). Importantly, ROC analysis showed that sera cELISA achieved a slightly superior AUC, indicating more consistent discrimination between infected and non-infected individuals. In addition, paired quantitative comparisons showed no significant difference between sera and DBS (Wilcoxon *p* = 0.22), and Bland–Altman analysis indicated minimal bias with moderately narrow limits of agreement, supporting the stability of quantitative differences between matrices. Taken together, these findings suggest that the higher sensitivity observed for DBS cELISA may reflect matrix-related analytical behavior rather than a true biological advantage. The sensitivity and specificity values obtained with the recombinant NIE antigen-based cELISA are consistent with those reported in previous studies [16,17,18].

A previous study also evaluated DBS for *S. stercoralis* serology and reported excellent concordance with serum (κ = 0.92). Importantly, their assay relied on crude antigen (Bordier ELISA test), whereas our evaluation used a recombinant NIE-ELISA [10]. These methodological differences—despite both being commercial tests—largely explain the contrast in kappa values between studies and indicate that matrix performance is assay-dependent.

In contrast, assays such as the NIE-ELISA are more sensitive to background and elution conditions, which likely explains the greater matrix-dependent variability observed in our setting. Thus, while [10] demonstrated the feasibility of DBS for field surveys, our results extend this evidence by showing that DBS compatibility is assay-dependent and that ELISAs using recombinant antigen may require specific optimization when applied to dried blood spots.

In contrast, the in-house NIE-ELISA exhibited a marked decline in performance when applied to DBS. The fair agreement (κ = 0.21) and reduced AUC (0.61) suggest that matrix effects, elution conditions, or antigen–antibody interactions may compromise assay stability. The DBS NIE-ELISA yielded an AUC of 0.61, markedly lower than the AUC obtained with DBS cELISA (0.83, *p* < 0.05). This indicates that the NIE-ELISA protocol requires further optimization for DBS matrices before it can be considered for diagnostic or epidemiological applications. Paired sera–DBS comparisons showed no significant difference (Wilcoxon *p* = 0.53), but the confidence interval of the median difference was wide, reflecting substantial variability. Bland–Altman analysis further demonstrated large dispersion between matrices, with a high bias and extremely wide limits of agreement, indicating inconsistent quantitative behavior of DBS for this assay. Our standardization experiments revealed that distilled water—the eluent recommended by the TropBio protocol—generated nonspecific reactivity in both ELISA platforms. Only the commercial diluent (cELISA) and PBS-milk (for NIE-ELISA) produced reproducible results, underscoring the importance of assay-specific optimization. These observations highlight a broader methodological principle: DBS are not universally compatible across serological assays, and their successful implementation requires careful validation of elution buffers, dilutions, and storage conditions.

Our findings also resonate with the previous study conducted in the same region using the SSrapid test [12], which demonstrated the feasibility of point-of-care serology for community-based screening. Together, these studies illustrate a continuum of diagnostic innovation—from rapid tests to DBS-based ELISA—tailored to the operational realities of endemic settings. DBS offer unique advantages: minimal invasiveness, simplified transport, and long-term stability, making them particularly suitable for large-scale surveys, remote communities, and integrated NTD programs.

This study has limitations. The absence of agar plate culture, one of the most sensitive parasitological methods, may have contributed to misclassification in the PRS. The storage time of DBS (up to 11 months) could have influenced antibody stability, although our results suggest that performance remained robust. Finally, the in-house NIE-ELISA requires further optimization before DBS can be recommended for this assay.

## 5. Conclusions

Our findings suggest that DBS-based cELISA is a promising and field-adapted approach for strongyloidiasis seroepidemiological screening in resource-limited settings. Its good sensitivity and the moderate-to-good agreement observed with serum indicate that DBS-cELISA may be useful for population-level surveillance, although it cannot yet be considered a validated diagnostic tool. Additional work is needed to evaluate operational feasibility, assess generalizability in other endemic contexts, and further refine DBS protocols—particularly for in-house assays—before broader implementation in public health programs can be recommended.

## Figures and Tables

**Table 1:** Test accuracy in the whole study population (Primary reference standard). PPV: Positive Predictive value; PPN: Negative Predictive Value.

Assay	True Positive/ Total TP	Sensitivity [95%CI]	True Negative/Total TN	Specificity [95%CI]	PPV [95%CI]	PPN [95%CI]
cELISA (Sera)	43/53	81.10% [68.0–90.5]	129/173	74.60% [67.4–80.9]	49.43% [42.3–56.5]	92.80% [88.0–95.8]
cELISA (DBS)	49/55	89.10% [77.7–95.9]	83/111	74.80% [65.6–82.5]	63.60% [55.6–70.9]	93.20% [86.6–96.7]
In-house NIE-ELISA (Sera)	38/53	71.70% [57.6–83.2]	121/173	69.90% [62.5–76.6]	42.20% [35.5–49.2]	89.00% [83.9–92.6]
In-house NIE-ELISA (DBS)	28/55	50.90% [37.1–64.6]	83/111	74.80% [65.6–82.5]	50.00% [39.8–60.1]	75.40% [69.7–80.4]

**Table 2:** Values of the ROC curve area for all methods.

Assay	AUC [95% CI]	*p*
cELISA_sera	0.85 [0.78–0.93]	<0.001
cELISA_DBS	0.83 [0.75–0.91]	<0.001
In house-NIE-ELISA_sera	0.78 [0.68–0.88]	<0.001
In house-NIE-ELISA_DBS	0.61 [0.50–0.73]	0.060

## Data Availability

The original contributions presented in this study are included in the article/Supplementary Material. Further inquiries can be directed to the corresponding authors.

## Supplementary Materials

The following supporting information can be downloaded at: https://www.mdpi.com/article/10.3390/pathogens15101049/s1, Figure S1: Optical density (450 nm) values obtained from DBS eluted using three diluents across different sample types.
